# Scenario analysis for potential community spread of Andes virus (ANDV)

**DOI:** 10.2807/1560-7917.ES.2026.31.22.2600425

**Published:** 2026-06-04

**Authors:** Valentina Marziano, Alfredo De Bellis, Martina Del Manso, Antonino Bella, Alberto Mateo Urdiales, Carla Molina Grané, Francesco Menegale, Lorenzo Lucchini, Mattia Manica, Sobha Pilati, Daniele Mipatrini, Flavia Riccardo, Giorgio Guzzetta, Piero Poletti, Anna Teresa Palamara, Patrizio Pezzotti, Stefano Merler

**Affiliations:** 1 Center for Health Emergencies, Fondazione Bruno Kessler, Trento, Italy; 2 Department of Infectious Diseases, Istituto Superiore di Sanità, Rome, Italy; 3 Department for Prevention, Research, and Health Emergencies, Ministry of Health, Rome, Italy; * These authors contributed equally to this work and share first authorship.; ** These authors contributed equally to this work and share last authorship.

**Keywords:** Hantavirus, Andes virus, mathematical modelling, transmission dynamics, risk assessment, scenario analysis

## Abstract

We simulated the potential community spread of Andes virus (ANDV) following the introduction of a single infectious individual in a generic population, based on epidemiological parameters derived from a human-to-human historical outbreak. Under current available evidence, our analyses suggest that, within 4 months from the index case’s symptom onset, the expected outbreak size is unlikely to exceed 50 cases, with a high probability of epidemic extinction, particularly when > 50% cases are effectively isolated from the start of the outbreak.

The 2026 cluster of severe respiratory illness associated with Andes virus (ANDV) detected on a cruise ship carrying passengers and crew from 23 countries raised concerns about its potential public health impact [1]. We analyse human-to-human ANDV transmission chain data collected during an outbreak of ANDV in Argentina in 2018–2019 [2] (hereafter ‘reference cluster’) to model the community spread of ANDV in a generic population, should the epidemic be left unmitigated or, alternatively, should cases be isolated. We estimate the expected epidemic size (i.e. number of cases) and the likelihood of outbreak extinction within a few months from the outbreak start.

## Andes virus key epidemiological parameters from historical data

We estimated the distribution of the serial interval (i.e. the time between symptom onset in an index case and symptom onset in a secondary case that got the infection from the index case), by fitting Gamma, log-normal, and Weibull distributions to serial intervals observed in the reference cluster [2]. The Gamma distribution provided the best fit according to the Akaike information criterion (AIC), yielding an estimated mean of 22.8 days (95% percentile interval: 11.3–38.4).

We estimated the individual-level heterogeneity in transmission, by fitting a negative binomial distribution to the offspring distribution observed in the reference cluster [2], considering only transmission events that occurred before the implementation of control measures. We constrained the mean of the distribution to the basic reproduction number R_0_ = 2.12 (median estimate reported in [2]) and calibrated the overdispersion parameter (*k*), finding *k* = 0.316, which indicates substantial transmission heterogeneity consistent with superspreading events [3]. We explored R_0_ values of 1.24 and 3.35, corresponding to the lower and upper bounds of the 95% credible interval (CrI) reported in [2], and re-estimated the corresponding overdispersion parameter values (*k* = 0.323 and *k* = 0.257, respectively).

## Community spread of Andes virus and the impact of isolation

We simulated the unmitigated ANDV community spread in a generic population (baseline scenario) using an individual-based stochastic model based on a branching process [4], assuming a single introduction of an infectious individual in a fully susceptible population. The contribution of asymptomatic and pre-symptomatic transmission was assumed to be negligible. Each infected individual generated a number of secondary infections according to a negative binomial distribution of mean R_0_ and overdispersion *k*. For each secondary infection generated, the delay between symptom onset in the infector and in the infectee was sampled from the estimated serial interval distribution. We ran simulations over a 120-day-time horizon, recording the full transmission tree as a case line list. To capture the stochastic variability of potential epidemic trajectories, we ran 1,000 independent model realisations. The time horizon was chosen to provide a comparable timeframe to the 2018–2019 reference cluster, which was controlled within 15 weeks.

**Figure 1:**
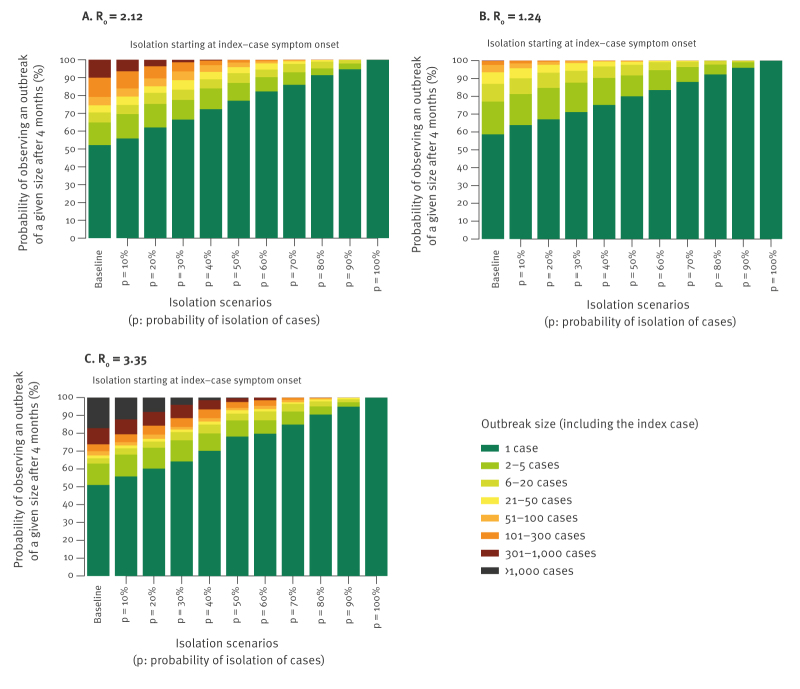
Impact of case isolation on the cumulative Andes virus outbreak size, depending on the transmission characteristics with (A) R_0_ = 2.12, (B) R_0_ = 1.24 and (C) R_0_ = 3.35

Results suggest that the previously estimated transmissibility (R_0_ = 2.12) combined with a relatively long serial interval (approximately 23 days) may lead to a very slow increase in case numbers during unmitigated outbreaks. In the unmitigated scenario, the total number of cases after 4 months was estimated below 50 in ca 75% of simulated outbreaks (Figure 1).

Probability of observing outbreaks with a given size within 120 days following symptom onset of the index case. Baseline: unmitigated transmission. Simulations are based on assumed parameter values of (A) R_0_ = 2.12 and overdispersion *k* = 0.316; (B) R_0_ = 1.24, *k* = 0.323; (C) R_0_ = 3.35, *k* = 0.257. Case isolation is implemented starting from the date of symptom onset of the first case. Percentages are calculated as the number of outbreaks out of n = 1,000 model realisations for each scenario.

We assessed the impact of different case isolation scenarios. We assumed that the intervention started T_I_ days after the index case’s symptom onset, with probability of isolation p (proportion of cases that are successfully identified and isolated at the time of their symptom onset), ranging from 10% to 100%. Starting from line lists in the unmitigated scenarios, each case having symptom onset after time T_I_ is isolated with probability p. For each isolated case, all linked subsequent generations of cases are removed from the line list, assuming no further onward transmission after isolation. In the main analysis, we set T_I_ = 0 (immediate interventions), representing transmission in a context where public health authorities are aware of the possibility of onward spread and have implemented measures to rapidly identify infections.

Early identification and case isolation can substantially reduce case numbers following outbreak detection (Figure 1). Under immediate interventions and assuming p > 50%, the cumulative number of cases after 4 months would unlikely exceed 50 (as the probability of an outbreak with less than 50 cases is greater than 95%) when R_0_ = 2.12. Even assuming R_0_ = 3.35, effectively isolating at least 70% of infections would likely keep the cumulative number of cases below 50 after 4 months (probability greater than 95%).

## Probability of epidemic extinction

We calculated, for each scenario, the probability that an outbreak generated from one single infected individual would die out within the observation horizon (120 days), using a probability-generating function (PGF) approach [5].

**Figure 2:**
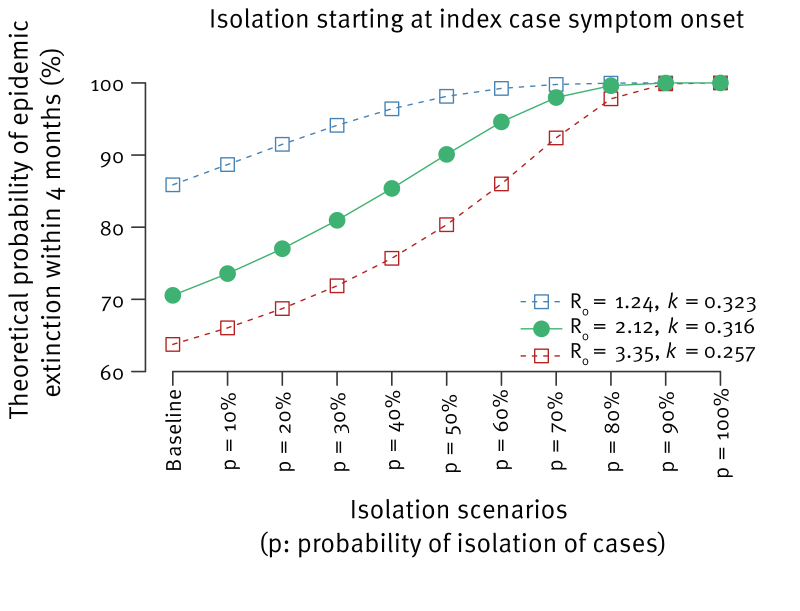
Probability of Andes virus epidemic extinction within 120 days following the symptom onset of the index case, as a function of isolation probability

We found that over 70% of simulated outbreaks reached extinction in unmitigated scenarios with R_0_ = 2.12 and over 60% with R_0_ = 3.35 (Figure 2). Transmission would die out within 4 months with a probability greater than 90% if p > 50% under R_0_ = 2.12, and p > 70% under R_0_ = 3.35.

Case isolation is implemented starting from the date of symptom onset of the first case.

## Sensitivity analyses

We ran two sensitivity analyses to assess the robustness of our results. We considered a scenario with case isolation implemented T_I_ = 30 days after the symptom onset of the index case, mimicking a delay in isolation of the first cases of the outbreak, as is likely to occur in the early phase of undetected outbreaks. In this case, the total number of cases after 4 months would stay within 50 with a probability greater than 95% if 80% of cases are effectively isolated when R_0_ = 2.12, as shown in the Supplement.

We considered a transmission scenario where no superspreading is assumed (i.e. the offspring distribution follows a Poisson with mean equal to R_0_). In this case, when R_0_ = 2.12, the total number of cases after 4 months was lower than 50 in around 45% of simulated outbreaks under unmitigated transmission and in over 95% of simulated outbreaks if 50% of cases were effectively isolated (see Supplement).

In the Supplement, we additionally report results considering two alternative time horizons, namely 90 and 180 days. Full methodological details and additional results are reported in the Supplement.

## Public health relevance

On 6 and 8 May 2026, the European Centre for Disease Prevention and Control (ECDC) and the World Health Organization (WHO) issued risk assessments, pointing out that the risk of ANDV spreading in the general population is low to very low [6,7]. Overall, our results are in line with international assessments indicating a low or very low risk of community spread, provided that cases can be effectively identified and isolated. Although our results point towards a scenario of slow and limited number of cases after several months of unmitigated transmission, the high case fatality ratio associated with ANDV in specific settings (20–50%) [2,8] warrants the implementation of maximum precautions to limit the infection spread. Our findings highlight that timely diagnosis and isolation of cases are key measures to interrupt ANDV community spread.

## Discussion

Model estimates suggest that the estimated transmissibility and long serial interval may lead to slow case growth following a single ANDV introduction in a susceptible population, while prompt case isolation can limit onward transmission and leads to a high likelihood of epidemic extinction. The capacity for case isolation depends on a pathogen’s natural history and the epidemiological context. During early severe acute respiratory syndrome coronavirus 2 (SARS-CoV-2) epidemics, characterised by rapid spread and silent transmission, contact tracing detected 31% of cases in Colombia [9], 53% in Singapore [10], and 98% in South Korea [11]. Current evidence highlights that ANDV infections are mostly symptomatic and severe, while longer incubation periods and serial intervals grant authorities more time to trace case contacts before they become infectious. As the current outbreak originated within the closed population of a cruise ship, exposed individuals were precisely identifiable and, to date, all subsequent cases were among people onboard [12]. Considering these epidemiological traits, achieving a high isolation proportion represents a feasible target for European countries.

The following limitations should be considered. Estimates of R_0_, the serial interval, and the degree of superspreading were either directly obtained or inferred from a single reference cluster [2]. The reported results critically depend on these parameter values. These estimates are based on a limited number of observed infections and are therefore subject to substantial uncertainty. Nevertheless, to the best of our knowledge, they currently represent the most reliable evidence available on human-to-human transmission of ANDV. The data currently available on the 2026 outbreak provide limited information for updating our epidemiological understanding of these parameters, especially since they refer to a transmission setting — cruise ships — that differs substantially from transmission in the general population. Given the ongoing epidemiological investigations and the limited evidence currently available, the occurrence of asymptomatic infections and their contribution to transmission dynamics remain uncertain and have not been considered in this preliminary assessment. Should asymptomatic and pre-symptomatic ANDV transmission [13] occur, population-level transmission risks may have been underestimated, and the effectiveness of case isolation could be overestimated, requiring a higher proportion of cases to be isolated to achieve comparable epidemic size reduction and extinction probabilities.

The international spread observed during the early phase of the outbreak suggests that transmission patterns involving multiple simultaneous transmission chains may result in epidemic dynamics that are more complex than those represented in this preliminary assessment, where we consider a single infectious index case. Model limitations include the assumption of perfect isolation, which ignores potential delays between the onset of infectiousness in a case and the time of its isolation, and the possibility of secondary transmission while in isolation. It should be emphasised that the results presented here represent scenario analyses and should not in any way be interpreted as predictions of the future course of the epidemic. Further epidemiological assessments will benefit from accumulating evidence on epidemiological parameters and on the potential contribution of asymptomatic infections and pre-symptomatic transmission to ANDV spread.

## Conclusion

Our analyses indicate that, within 4 months of symptom onset in the index case of ANDV infection, any resulting outbreak would be unlikely to exceed 50 cases. We also show that rapid case detection and effective isolation can considerably reduce onward transmission and increase the likelihood of outbreak extinction. The results of this analysis align with international assessments that identify a low or very low risk of transmission of ANDV in the general population. With further epidemiological knowledge of ANDV transmission, our approaches and estimates can potentially be improved and refined.

## Data Availability

All data generated or analysed during this study are included in this article and the Supplement.

## Supplementary Data

381000 Supplementary Material
