# Supplementary material for: Scenario analysis for potential community spread of Andes virus (ANDV)
Source: Euro Surveill. 2026 Jun 4;31(22):2600425. doi: 10.2807/1560-7917.ES.2026.31.22.2600425 (PMC13241788; doi:10.2807/1560-7917.ES.2026.31.22.2600425)
Supplement: Supplementary Material [file 26-00425_PEZZOTTI_Supplement.pdf]

Supplement

Disclaimer: This supplementary material is hosted by Eurosurveillance as supporting information alongside the article "Scenario Analysis for Potential Community Spread of Andes Virus (ANDV)", on behalf of the authors, who remain responsible for the accuracy and appropriateness of the content. The same standards for ethics, copyright, attributions and permissions as for the article apply. Supplements are not edited by Eurosurveillance and the journal is not responsible for the maintenance of any links or email addresses provided therein.

Data source

We used epidemiological data from the reconstructed transmission chains reported by Martínez et al. [1] for the 2018-2019 human-to-human outbreak of Andes virus (ANDV) hantavirus pulmonary syndrome in Chubut Province, Argentina. The outbreak included 34 confirmed cases and 11 deaths. Transmission links were reconstructed by Martínez et al. using clinical and epidemiological information, contact tracing, serological testing, and next-generation sequencing.

For the present analysis, we used the reconstructed transmission chains to parameterize two key epidemiological quantities relevant for potential community spread: the serial interval distribution and the offspring distribution. Following Martínez et al., we retained all epidemiological links classified as "certain" or "likely", yielding 33 infector-infectee pairs.

Serial interval distribution

We estimated the ANDV serial interval distribution, defined as the time between symptom onset in an infector and symptom onset in the corresponding infectee.

We fitted three candidate parametric distributions to the observed serial intervals using maximum likelihood estimation (MLE): Gamma, log-normal, and Weibull distributions. Model fit was compared using the Akaike information criterion (AIC). The Gamma distribution provided the best fit to the data and was therefore used in the scenario analysis (see Table S1).

Table S1. Comparison of candidate parametric distributions for the ANDV serial interval.

Distribution	AIC	Log-likelihood
**Gamma**	216.79	-106.39
**Log-normal**	217.36	-106.68
**Weibull**	219.44	-107.72

The fitted Gamma distribution had shape parameter 10.77 and scale parameter 2.12, corresponding to a mean serial interval of 22.8 days and a 95% percentile interval of 11.3-38.4 days, yielding consistent estimates with those provided in the original study. The fitted distribution is shown in Figure S1.

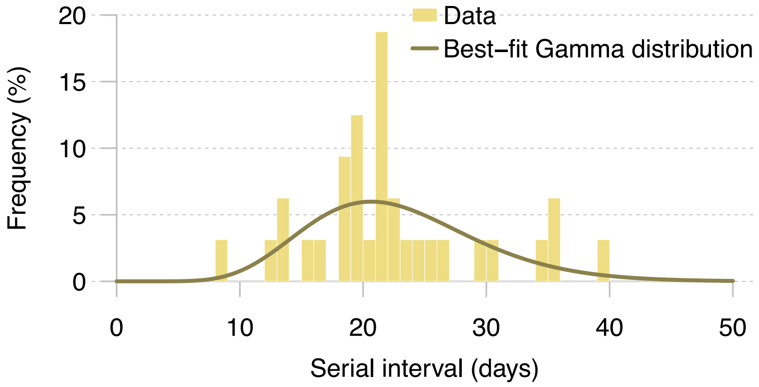

Figure S1. Estimated serial interval distribution for ANDV. Bars show the observed serial intervals obtained from 33 infector-infectee pairs classified as "certain" or "likely" in the reconstructed transmission chains reported by Martínez et al. [1]. The solid line shows the best-fitting Gamma distribution estimated by maximum likelihood. Estimated parameters: shape=10.77; scale=2.12.

Individual heterogeneity in transmission and superspreading

To characterize heterogeneity in individual transmission, and the potential occurrence of superspreading events, we derived the observed offspring distribution, defined as the number of secondary cases generated by each infected individual, from the reconstructed transmission chains.

During the 2018-2019 outbreak, public health interventions, including isolation of confirmed cases and quarantine of contacts, were introduced in early 2019, approximately two months after the start of the outbreak.

Because control measures substantially altered transmission dynamics, in line with the approach adopted by Martinez et al., we restricted our analysis to the pre-control phase of the outbreak. Specifically, we retained 28 transmission events over 18 distinct infectors, occurring entirely before the implementation of isolation and quarantine measures. We fitted a negative-binomial offspring distribution [2], with the mean of the distribution fixed to the values of the pre-control reproduction number estimated by Martínez et al. (R_0=2.12, 95% Credible Interval: 1.24-3.35), while the overdispersion parameter k was estimated from the observed offspring distribution using MLE. We obtained k=0.316 when considering R_0=2.12, k=0.257 when considering R_0=1.24 and k=0.323 when considering R_0=3.35 respectively. These estimates imply substantial superspreading, with most infected individuals generating few or no secondary cases and a small number of infections accounting for a disproportionate share of onward transmission [2]. The fitted offspring distribution is shown in Figure S2 for R_0=2.12 and k=0.316.

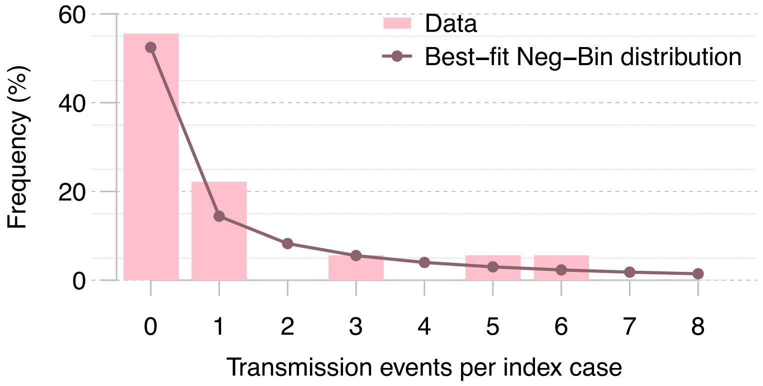

Figure S2. Distribution of the number of secondary cases generated by each infector. Bars show the observed distribution of the number of secondary cases generated by infected individuals during the pre-control phase of the 2018-2019 ANDV outbreak. The solid line shows the fitted negative-binomial distribution, obtained by fixing the mean reproduction number at R_0=2.12 and estimating the overdispersion parameter k. The estimated value was k=0.316.

Transmission model in the absence of interventions

To assess the potential epidemic spread of ANDV, we used an individual-based stochastic model based on a branching process [3]. Specifically, transmission dynamics was modeled as a discrete stochastic transmission tree, in which each

infected individual can generate a random number of secondary cases according to a negative-binomial distribution. Model simulations were initialized with a single index case with symptom onset on day 1. For each infected individual i, the number of secondary infections Z_i was sampled from a negative-binomial distribution parameterized by a mean reproduction number R_0 and an overdispersion parameter k:

$$\mathrm{Z}_i \sim \mathrm{NegBin}(\mathrm{R}_0, k)$$

We considered the following pairs of values: R_0=2.12, k=0.316; R_0=1.24, k=0.323; and R_0=3.35, k=0.257.

For each secondary case $j \in \{1, \dots, \mathrm{Z}_i\}$ generated by a primary case i, the delay between symptom onset in the infector and in the infectee, corresponding to the serial interval, was sampled from a Gamma distribution:

$$\sigma_j \sim \Gamma(\alpha, \theta)$$

with shape parameter α=10.77 and scale parameter θ=2.12 (see section “Serial interval distribution”).

The symptom onset time t_j was therefore defined as:

$$t_j = t_i + \mathrm{round}(\sigma_\mathrm{j})$$

where time was represented in integer days.

To capture the stochastic variability of potential epidemic trajectories, we simulated N=1000 independent model realizations. For each realization, the full transmission tree was recorded as a case line list, including the case identifier, the identifier of the corresponding infector, and the symptom onset time of each case. Each epidemic realization was simulated over a 120-day time horizon starting from day 1, corresponding to the symptom onset date of the index case. Secondary cases whose simulated symptom onset time exceeded this time horizon were discarded and excluded from the transmission tree.

In a sensitivity analysis, we assumed that ANDV transmission is not characterized by superspreading; accordingly, Z_i is sampled from a Poisson distribution with mean R_0:

$$\mathrm{Z}_i \sim \mathrm{Poisson}(\mathrm{R}_0)$$

In the Additional results section, we report results considering two alternative time horizons, namely 90 days and 180 days.

Potential impact of case isolation interventions

The potential impact of case isolation interventions was assessed as follows. For each epidemic realization obtained under the unmitigated scenario, we simulated an isolation intervention implemented from time T_I since the symptom onset of the index case by pruning the downstream transmission trees of all isolated individuals. In the main analysis, we considered T_I=0; in a sensitivity analysis, we considered T_I=30 days. We explored different values for the probability of isolation, p, ranging from 10% to 100% in increments of 10%. This probability was defined as the proportion of cases that would be successfully identified and isolated at the time of symptom onset.

Specifically, for each epidemic realization (N=1000) and isolation probability p, the implemented algorithm worked as follows:

1) For all cases with symptom onset after T_I, isolation status was assigned probabilistically with probability p. Cases with symptom onset before the intervention were not isolated. This assumption reflects the observation by [1] that 89% of infectors for which the information was known (17/19) transmitted the virus within the first 24 hours from symptom onset, and the remaining ones within 3 days.
2) For each isolated case, we removed the entire transmission chain originating from that case. This reflects the assumption that isolated cases can no longer transmit the infection to other individuals.

This approach allowed us to estimate the counterfactual epidemic size that would occur if isolation interventions were implemented at different levels, while preserving the stochastic realizations of unmitigated epidemics.

Probability of epidemic extinction

We calculated the probability that an epidemic would die out by the end of the observation horizon (120 days in the main analysis) using an analytical approach that complements numerical simulations by providing exact probabilities within the assumed transmission model. For each value of R_0 and k, we calculated the theoretical extinction probability using a probability-generating function (PGF) approach [4].

The central idea is that an epidemic becomes extinct only if all transmission chains generated by each infected individual die out. Let u denote the symptom onset time of an infected individual, and assume that the mean number of secondary cases, $\mu(u)$, may vary with u. If an individual with symptom onset at time u generates z secondary cases, the epidemic becomes extinct by the final observation time T only if all z downstream secondary transmission chains become extinct by T. For a single secondary case occurring after a delay τ, this requires the chain generated by that case to become extinct within the remaining time interval, from $u+\tau$ to T. Therefore, the probability that any secondary chain becomes extinct by time T is:

$$\mathrm{A}(u;\mathrm{T}) = \int_0^{\mathrm{T}-u} \sigma(\tau)\mathrm{H}(u+\tau;\mathrm{T})\mathrm{d}\tau$$

where $\sigma(\tau)$ is the serial interval distribution and $\mathrm{H}(u;\mathrm{T})$ is the probability that the entire transmission chain generated by a case with symptom onset at time u becomes extinct by time T. If the individual generates exactly z secondary cases, and if the subchains are independent, the probability that all of them become extinct by T is $\mathrm{A}(u;\mathrm{T})^z$. Since the number of secondary cases is stochastic, we average this quantity over the distribution of the number of secondary cases:

$$\mathrm{H}(u;\mathrm{T}) = \sum_{z=0}^{\infty} \mathrm{P}(\mathrm{Z}_u = z)\mathrm{A}(u;\mathrm{T})^z$$

This sum is exactly the PGF of the offspring distribution, evaluated at $\mathrm{A}(u;\mathrm{T})$, namely:

$$\mathrm{H}(u;\mathrm{T}) = \mathrm{G}_u\big(\mathrm{A}(u;\mathrm{T})\big)$$

If the distribution of the number of secondary cases is negative-binomial with mean $\mu(u)$ and overdispersion parameter k, the PGF is:

$$\mathrm{G}_u(s) = \left[1 + \frac{\mu(u)}{k}(1-s)\right]^{-k}$$

therefore:

$$\mathrm{H}(u;\mathrm{T}) = \left[1 + \frac{\mu(u)}{k}\left(1 - \int_0^{\mathrm{T}-u} \sigma(\tau)\mathrm{H}(u+\tau;\mathrm{T})\mathrm{d}\tau\right)\right]^{-k}$$

The probability that the epidemic generated by the index case becomes extinct by time T is therefore $\mathrm{H}(0;\mathrm{T})$. When isolation of symptomatic cases is active from the start of the epidemic, $\mu(u) = (1-p)\mathrm{R}_0$ for all u, so the equation reduces to the time-homogeneous formulation, depending only on the remaining time. If isolation is activated only after a delay T_I, we use $\mu(u) = \mathrm{R}_0$ for $u < \mathrm{T}_\mathrm{I}$ and $\mu(u) = (1-p)\mathrm{R}_0$ for $u \geq \mathrm{T}_\mathrm{I}$. We numerically evaluated the solution of this equation for T=120 days (4 months) from symptom onset in the index case, and for T_I=0 or 30 days.

Specifically, we solved this equation directly by backward recursion by discretizing the interval $[0,\mathrm{T}]$ with a time step of 0.1 days. The serial interval distribution was converted into discrete probability masses over the corresponding time bins, and $\mathrm{H}(u;\mathrm{T})$ was computed by backward recursion from the final observation time $u = \mathrm{T}$ to the index-case onset time $u = 0$. At the final time, extinction by T requires no future secondary infections, so we set $\mathrm{H}(\mathrm{T};\mathrm{T}) = \mathrm{G}_\mathrm{T}(0) =$

$\left(1+\frac{\mu(u)}{k}\right)^{-k}$. For each earlier time point, the integral over the serial interval distribution was solved numerically using the values of H at later times that had already been computed.

We also solved the same equation using the different values of R_0 and overdispersion considered. As sensitivity analysis, we repeated the calculations assuming a Poisson offspring distribution and shorter or longer time horizons (90 and 180 days).

Additional results

Figures S3 and S4 report the sensitivity analysis in which isolation was assumed to begin 30 days after symptom onset of the index case, rather than immediately at start of the outbreak. This scenario may be illustrative of conditions associated with the initial phase of the 2026 outbreak.

Assuming R_0=2.12, we obtain that the probability of having less than 50 cases within 4 months is greater than 90% if at least 80% of cases are effectively isolated. Assuming R_0=1.24, the probability of having less than 50 cases within 4 months is greater than 90% even in the absence of isolation (baseline scenario). On the other hand, when assuming R_0=3.35, the probability of having less than 50 cases within 4 months is greater than 90% only when assuming that 100% of cases are effectively isolated.

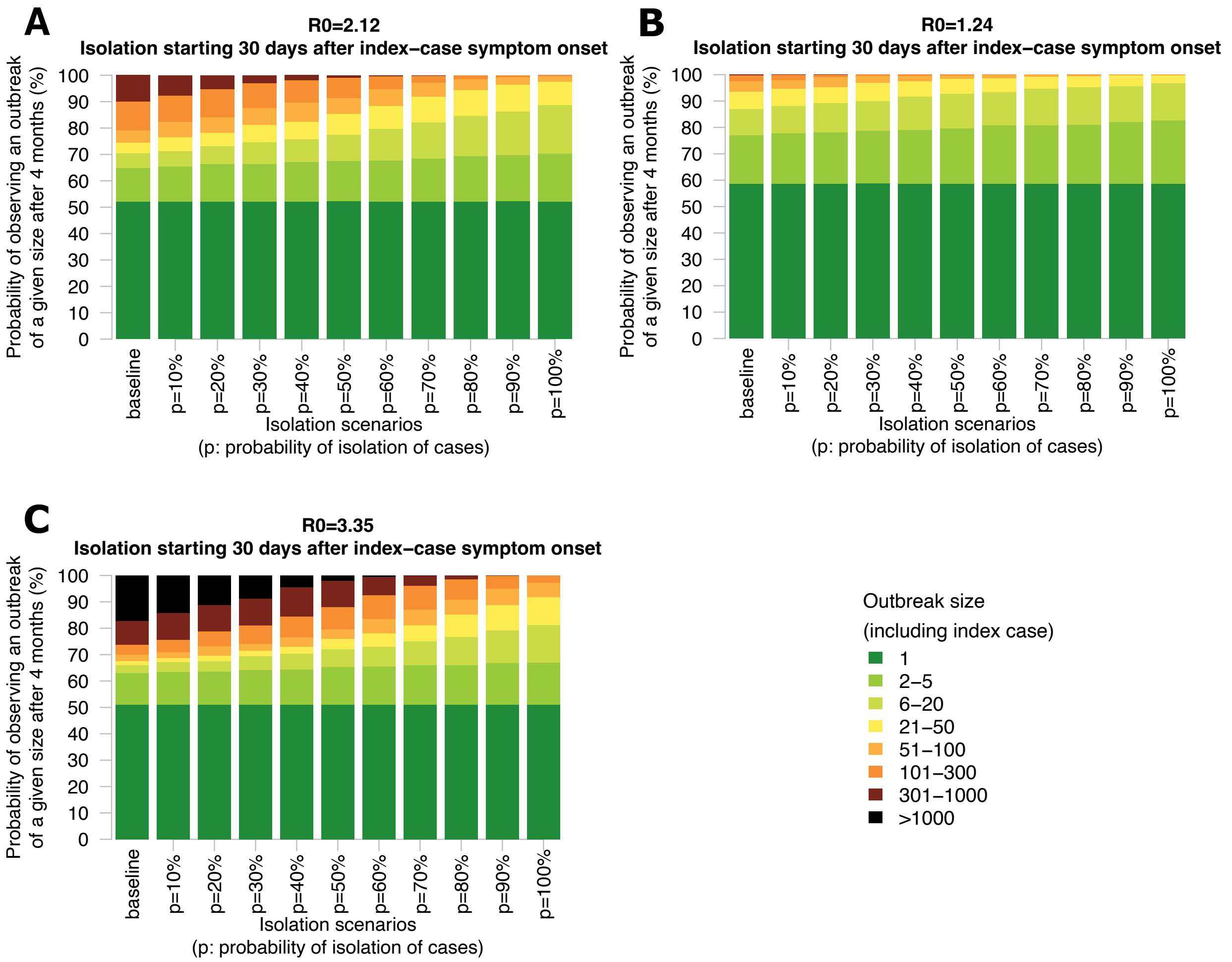

Figure S3. Impact of case isolation on the cumulative outbreak size for different values of $\mathbf{R_0}$. Probability of observing outbreaks with a given size within 120 days following symptom onset of the index case. Case isolation is implemented starting 30 days after the date of symptom onset of the index case. Simulations are based on the following assumed parameter values A) R_0=2.12 and overdispersion k=0.316; B) R_0=1.24 and overdispersion k=0.323; C) R_0=3.35 and overdispersion k=0.257. Percentages are calculated as the number of outbreaks out of N=1000 epidemic realizations for each scenario.

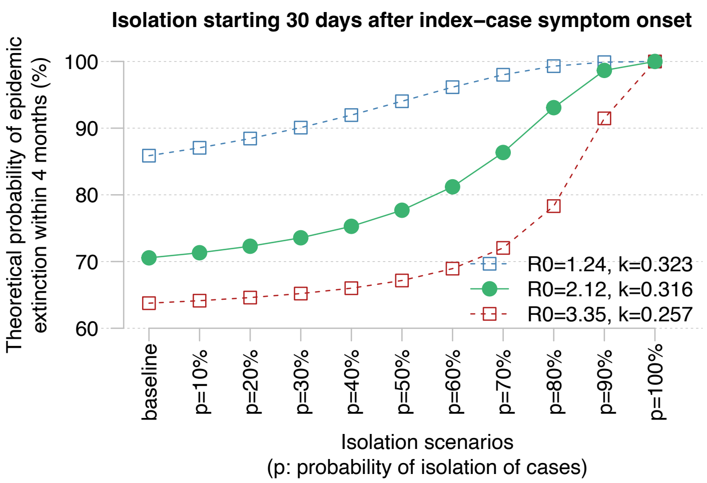

Figure S4. Probability of epidemic extinction within 120 days following the symptom onset of the index case, as a function of isolation probability. Isolation begins 30 days after the start of the outbreak. The extinction probability was calculated using the recursive probability generating function approach for the branching process (as described in the section "Probability of epidemic extinction"). The points and the solid green line show the baseline scenario R_0=2.12, while the points and dashed lines show the sensitivity analyses with R_0=1.24 (blue) and R_0=3.35 (red).

Figures S5 and S6 report a sensitivity analysis in which the offspring distribution was assumed to be Poisson with rate R_0 rather than negative-binomial. This corresponds to a scenario with no superspreading.

In this sensitivity analysis, assuming R_0=2.12, we obtain that the probability of having less than 50 cases within 4 months is greater than 90% if at least 40% of cases are effectively isolated. Assuming R_0=1.24, the probability of having less than 50 cases within 4 months is greater than 90% even in the absence of isolation (baseline scenario). Assuming R_0=3.35, we obtain that the probability of having less than 50 cases within 4 months is greater than 90% if at least 60% of cases are effectively isolated.

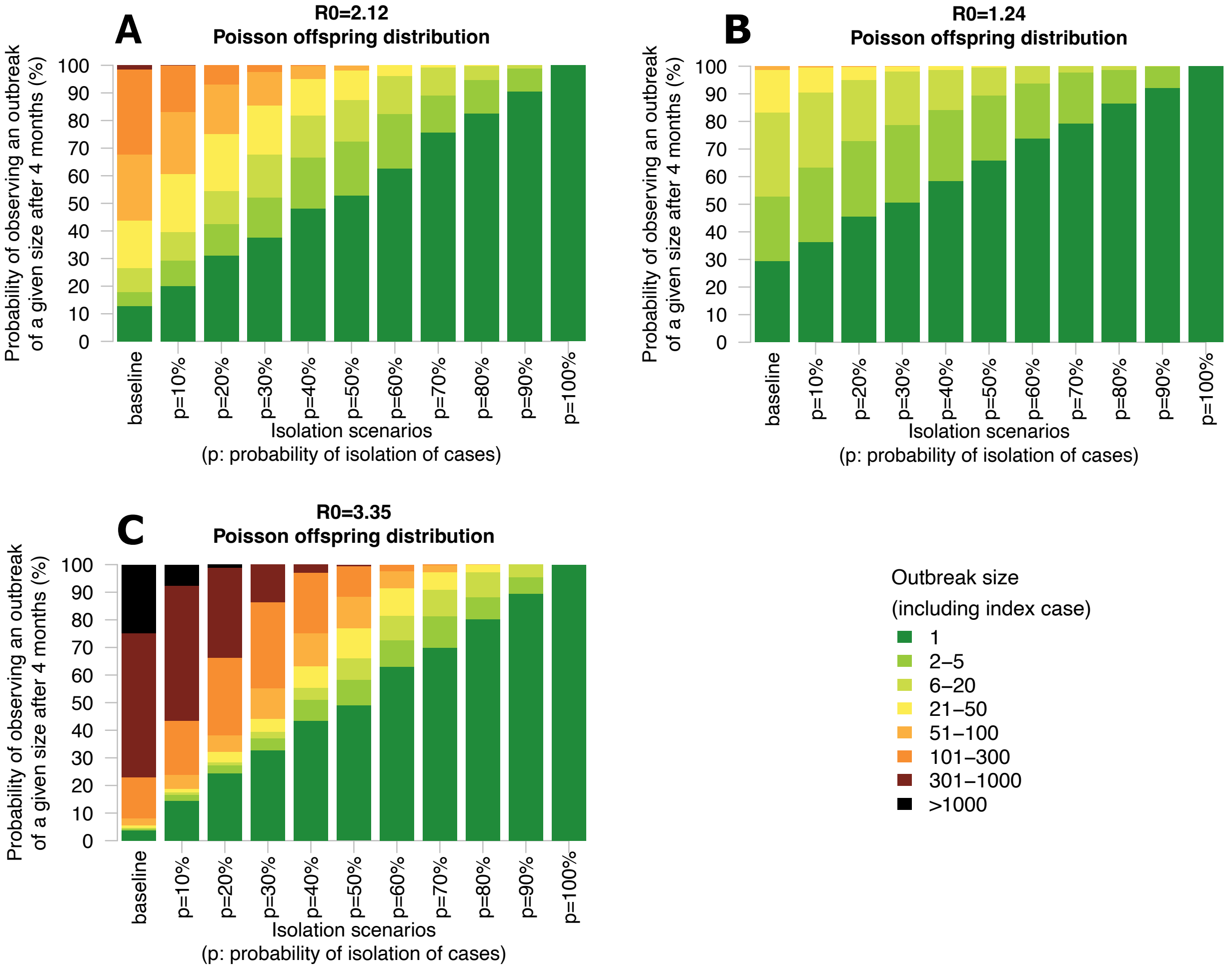

Figure S5. Impact of case isolation on the cumulative outbreak size for different values of $\mathbf{R_0}$. Probability of observing outbreaks with a given size within 120 days following symptom onset of the index case. The offspring distribution follows a Poisson distribution with mean R_0. Simulations are based on the following assumed parameter values A) R_0=2.12 and overdispersion k=0.316; B) R_0=1.24 and overdispersion k=0.323; C) R_0=3.35 and overdispersion k=0.257. Case isolation is implemented starting from the date of symptom onset of the index case. Percentages are calculated as the number of outbreaks out of N=1000 epidemic realizations for each scenario.

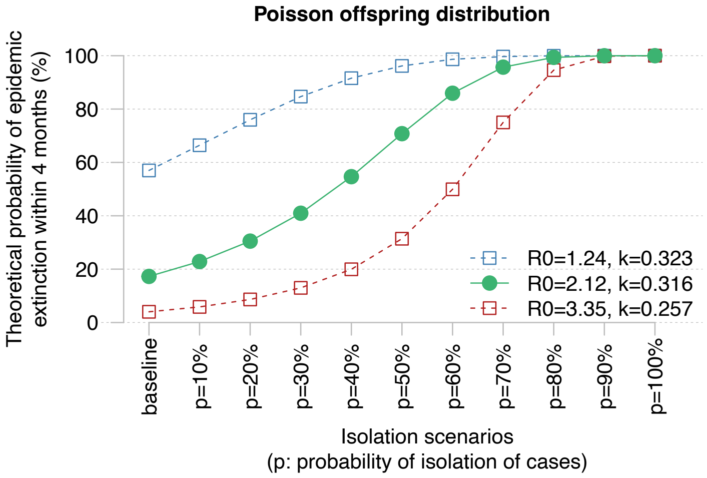

Figure S6. Probability of epidemic extinction within 120 days following the symptom onset of the index case, as a function of isolation probability. The offspring distribution follows a Poisson distribution with mean R_0. Isolation is implemented starting from the date of symptom onset of the index case. The extinction probability was calculated using the recursive probability generating function approach for the branching process (as described in the section “Probability of epidemic extinction”). The points and the solid green

line show the baseline scenario R_0=2.12, while the points and dashed lines show the sensitivity analyses with R_0=1.24 (blue) and R_0=3.35 (red).

Figure S7 and S8 show results obtained when considering a time horizon of 90 days or 180 days (and the median estimate of R_0=2.12). We obtain that the probability of having less than 50 cases within 3 months is greater than 90% if at least 30% of cases are effectively isolated. On the other hand, the probability of having less than 50 cases within 6 months is greater than 90% if at least 40% of cases are effectively isolated.

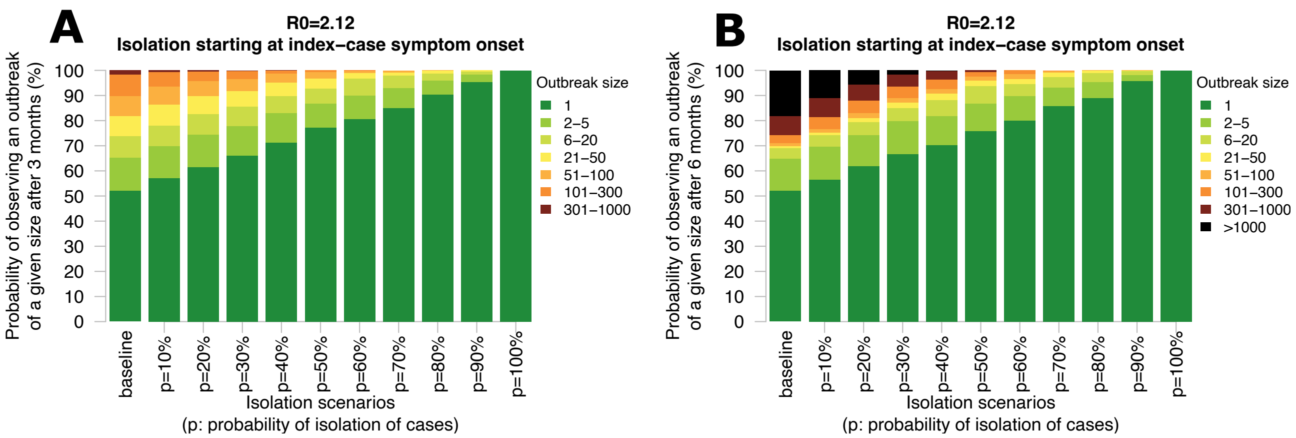

Figure S7. Impact of case isolation on the cumulative outbreak size when considering different time horizons. Probability of observing outbreaks with a given size within A) 90 days (3 months) and B) 180 days (6 months) following symptom onset of the index case. The offspring distribution follows a Negative Binomial distribution with mean R_0=2.12 and overdispersion k=0.316. Case isolation is implemented starting from the date of symptom onset of the index case. Percentages are calculated as the number of outbreaks out of N=1000 epidemic realizations for each scenario.

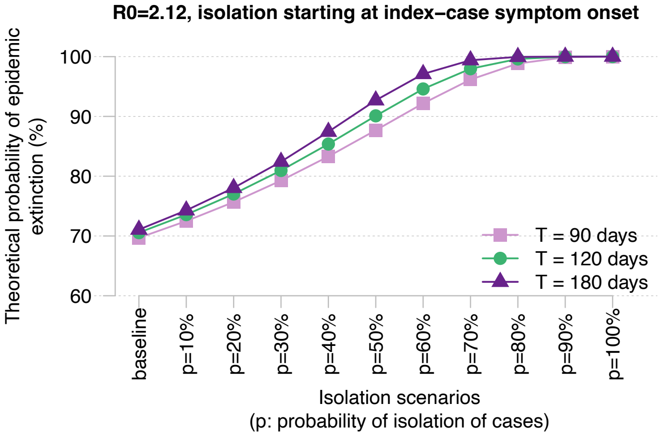

Figure S8. Probability of epidemic extinction within 90, 120 and 180 days following the symptom onset of the index case, as a function of isolation probability. The offspring distribution follows a Negative Binomial distribution with mean R_0=2.12 and overdispersion k=0.316. Isolation is implemented starting from the date of symptom onset of the index case.

To validate the analytical results obtained through the probability-generating function (PGF) approach, we compared the theoretical extinction probabilities with extinction probabilities computed from the 1000 stochastic model realizations. Specifically, we calculated the proportion of simulated outbreaks that remained below different cumulative size thresholds (≤5, ≤10, ≤15, ≤20, ≤25) within the 120-day time horizon. As shown in Figure S7, the probabilities of extinction computed from the stochastic simulations closely match the one obtained with the PGF approach across all isolation scenarios.

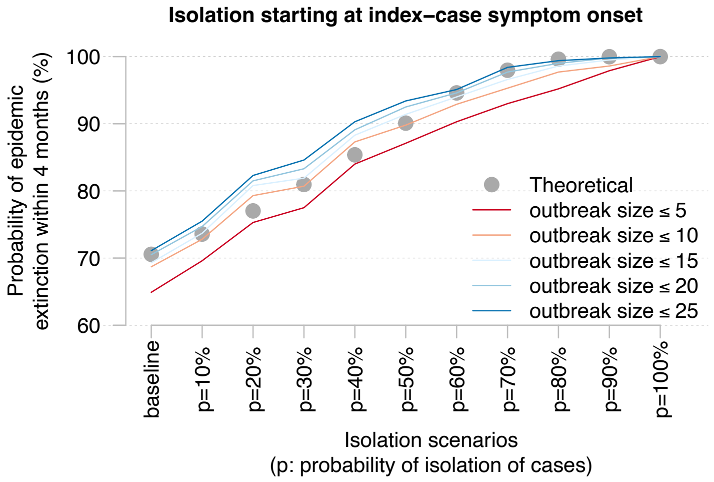

Figure S9. Validation of extinction probabilities (R_0=2.12 and T_I=0). Comparison between theoretical extinction probability calculated through the PGF approach (grey line) and the proportion of 1,000 simulated outbreaks that resulted in a cumulative size below various thresholds within 120 days (coloured lines).

References

[1] Martínez VP, Di Paola N, Alonso DO, Pérez-Sautu U, Bellomo CM, Iglesias AA et al. "Super-Spreaders" and Person-to-Person Transmission of Andes Virus in Argentina. N Engl J Med. 2020;383(23):2230-2241.

[2] Lloyd-Smith J, Schreiber S, Kopp P, Getz WM. Superspreading and the effect of individual variation on disease emergence. Nature. 2005;438:355–359.

[3] Haccou P, Jagers P, Vatutin VA. Branching processes: variation, growth, and extinction of populations. Cambridge university press; 2005.

[4] Southall E, Ogi-Gittins Z, Kaye AR, Hart WS, Lovell-Read FA, Thompson RN. A practical guide to mathematical methods for estimating infectious disease outbreak risks. Journal of Theoretical Biology. 2023;562:111417.
